# Harnessing the Nutritional Value, Therapeutic Applications, and Environmental Impact of Mushrooms

**DOI:** 10.1002/fsn3.70611

**Published:** 2025-07-15

**Authors:** Solomon Fitsum, Desta Berhe Sbhatu, Gebreselema Gebreyohannes

**Affiliations:** ^1^ Department of Biological and Chemical Engineering Mekelle Institute of Technology, Mekelle University Mekelle Ethiopia

**Keywords:** bioactive compounds, bioremediation, cosmetic, food, mushrooms, therapeutic properties

## Abstract

Mushrooms have been valued for their unique flavor and medicinal properties since ancient times. As a nutrient‐dense superfood, they provide essential nutrients, including dietary fiber, high‐quality protein, and key vitamins (especially D and B), along with minerals. They are naturally low in fat, sugar, and calories, and cholesterol, making them a beneficial addition to a balanced diet. Increasingly, mushrooms are incorporated into functional foods, cosmetics, and even used as meat substitutes, enhancing nutritional value and offering significant health benefits. They contain a variety of bioactive compounds—such as terpenoids, flavonoids, tannins, alkaloids, polyphenols, and polysaccharides—which contribute to their therapeutic properties. These include antioxidant, anticancer, antimicrobial, immunomodulatory, anti‐inflammatory, anti‐tyrosinase, immune‐boosting, and antihyperglycemic effects, all of which support human health and medical applications. Additionally, mushrooms promote heart health, help reduce cholesterol, and assist in managing chronic conditions like hypertension, obesity, and diabetes. Ongoing research continues to uncover new bioactive compounds that may be effective in treating neurological and cardiovascular diseases. With advancements in science and technology, mushrooms are becoming increasingly valuable in medicine, pharmaceuticals, food production, cosmetics, and environmental bioremediation, creating potential opportunities across various industries. This review aims to explore the nutritional value and bioactive compounds of mushrooms, along with their health benefits, therapeutic properties, and applications in functional foods, cosmetics, and pharmaceuticals. It highlights their potential as sustainable medicinal resources and a rich source of novel bioactive compounds for managing chronic diseases and other health conditions.

## Graphical abstract

Mushrooms are highly nutritious, packed with vitamins, minerals, antioxidants, essential amino acids, and bioactive compounds. They offer various health benefits, including boosting immunity, reducing inflammation, and supporting gut and cardiovascular health. Rich in carbohydrates, quality protein, essential fatty acids, and both soluble and insoluble fibers, they are versatile for culinary use. Furthermore, mushrooms and their byproducts show promise in food enhancement, cosmetics, pharmaceuticals, and as a sustainable animal feed.

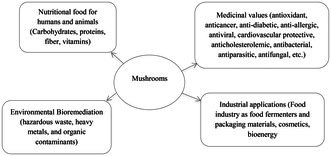

## 1 Introduction

Mushrooms (henceforth referred to as “they”) are renowned for their nutritional value and culinary diversity, making them a valuable addition to daily diets since ancient times (Bakratsas et al. 2021). They are a species of fungi belonging to the Basidiomycetes or Ascomycetes orders, and they are popular in both Eastern and Western countries due to their nutritional value, taste, and aroma. Additionally, they are high‐quality food products that can help underprivileged populations in developing countries overcome food and nutritional issues (Waktola and Temesgen 2018). They, traditionally used for health maintenance and disease prevention, are now being explored as mini‐pharmaceutical factories, capable of producing compounds with unique biological properties (Venturella et al. 2021).

Around 14,000 species of mushrooms exist, with 2000 considered edible and 200 commercially cultivated for Ayurvedic medicine and human consumption. They have been used for centuries for their health benefits, such as boosting the immune system, reducing inflammation, and promoting overall well‐being. Mushrooms are popular superfoods due to their nutritional value and unique flavors. They are rich in vitamins, minerals, and antioxidants, making them a valuable addition to a healthy diet. Their umami flavor adds savory depth to dishes, making them versatile and nutritious for those seeking natural health benefits (Das et al. 2022; Gebreyohannes et al. 2019a, 2019b). Edible mushrooms, commonly used in soups, tinctures, and teas, possess therapeutic properties and are utilized in biopharmaceutical applications. Due to their substantial health benefits, they are often compared to meat, fish, eggs, and dairy products, making them a valuable addition to both medicine and food processing (Elkhateeb et al. 2019; Kumar et al. 2021).

This review paper aimed to provide an in‐depth analysis of the nutritional value and diverse bioactive compounds found in mushrooms, highlighting their significant health benefits and potential therapeutic uses. These benefits stem from essential nutrients and potent compounds such as polysaccharides and polyphenols, which exhibit antioxidant, anti‐inflammatory, immunomodulatory, and neuroprotective effects, positioning mushrooms as promising resources for health promotion and disease prevention. Furthermore, this review delves into the multifunctional applications of mushrooms across the food, cosmetics, and pharmaceutical industries, where they enhance nutritional value, improve skincare, and contribute to the management of chronic diseases. Ultimately, it emphasizes the role of mushrooms as sustainable medicinal resources, offering eco‐friendly solutions and aligning with global efforts to develop innovative, natural therapeutic alternatives that promote well‐being while minimizing ecological impact.

## 2 Mushrooms Bioactive Compounds

They are gaining popularity due to their bioactive metabolites, which offer medicinal and health benefits, including disease prevention and treatment, as well as their nutritional value (Singh et al. 2025). Mushroom bioactive metabolites benefit human health by, for example, playing a crucial role in biotechnological processes like fermentation and extraction. They also show promise as dietary supplements for preventing and treating conditions such as cancer, cardiovascular disease, and diabetes. Their unique chemical structures and biological activities promote health through antioxidant mechanisms. As research continues to support these health‐promoting effects, the development of targeted dietary supplements presents a growing opportunity (Li et al. 2021).

Mushrooms are a rich source of bioactive compounds, including terpenoids, flavonoids, tannins, alkaloids, and polyphenols, all of which offer various health benefits. Terpenoids exhibit anti‐inflammatory properties, flavonoids support cardiovascular health, tannins possess antimicrobial effects, alkaloids demonstrate potential analgesic and psychoactive properties, and polyphenols are known to reduce the risk of chronic diseases (Bhambri et al. 2022). Additionally, mushrooms contain lectins and enzymes that enhance cell signaling and immune responses, potentially aiding in infection prevention. Enzymes, essential for digestion and metabolism, are found in the fruiting bodies, secondary metabolites, and cellular components (Phillips et al. 2022).

High‐protein foods are beneficial for heart patients and adults because they are low in calories and rich in water, minerals, and essential vitamins. However, their nutrient concentration and effectiveness can vary depending on factors such as food type, substrate, fruiting body, developmental stage, freshness, storage conditions, and cooking methods. These functional foods are especially advantageous for diabetics and individuals trying to lose excess fat due to their low starch content (Assemie and Abaya 2022; Singh et al. 2025).

## 3 Applications of Mushrooms in Food Enhancement

### 3.1 Mushrooms as a Food

Mushrooms, a staple in human diets since ancient times, provide a unique taste and significant nutritional value. Rich in umami, vitamins, minerals, and bioactive compounds, they contribute to overall health. Available in fresh, dried, or powdered forms, mushrooms accommodate diverse dietary preferences and cooking styles, helping to promote a balanced diet (Wang and Zhao 2023). Mushrooms are a highly nutritious superfood, packed with protein, essential amino acids, vitamins, and minerals. They are low in fat and sugar while being an excellent source of fiber, offering significant health benefits such as supporting diabetes management, promoting heart health, relieving constipation, and addressing vitamin D deficiency (Cardwell et al. 2018; Mallikarjuna et al. 2012).

#### 3.1.1 Mushrooms as Sources of Carbohydrates

Mushrooms are rich in carbohydrates, accounting for 50%–65% of their composition. These carbohydrates are primarily oligosaccharides and monosaccharides, with various derivatives contributing to their properties. Oligosaccharides promote gut bacteria growth and digestive health, while monosaccharides provide energy. Polysaccharides, like β‐glucans, enhance immune function and lower cholesterol levels (Table 1). Mushrooms contain both low and high molecular weight carbohydrates, including primary sugars like mannitol, trehalose, glucose, sucrose, fructose, raffinose, xylose, mannan, pectin, glucan, and glycogen (Zhou et al. 2024). The proportion of carbohydrates in mushrooms varies across growth phases and is distributed differently in their fruiting bodies, including the pileus, base, and stipe (Zhou et al. 2016).

**TABLE 1:** Proximate composition of some widely cultivated mushrooms (dry mass basis).

Mushroom species	Proximate composition (%)						References
	P	F	Carb	F	A	E (Kcal/kg)	
*A. bisporus*	14.1	2.2	74.0	12.2	9.7	325	(Sileshi et al. 2023)
*Lentinus edodes*	4.5	1.73	87.1	12.9	6.7	722	(Sileshi et al. 2023)
*Pleurotus ostreatus*	7.0	1.4	85.9	14.1	5.7	416	(Sileshi et al. 2023)
*Pleurotus eryngii*	11.0	1.5	6.2	81.4	6.2	421	(Sileshi et al. 2023)
*Pleurotus sajor‐caju*	37.4	1.0	55.3	9.8	6.3	—	(Sileshi et al. 2023)
*Pleurotus giganteus*	17.7	4.3	78.0	—	—	364	(Sileshi et al. 2023)
*Volvariella volvacea*	2.4	—	3.2	1.6	—	16	(Huang et al. 2022)
*Hypsizigus marmoreus*	2.11	0.35	3.87	1.3	3.1	22	(Huang et al. 2022)
*Flammulina velutipes*	2.66	0.29	7.81	2.7	21.5	37	(Yu et al. 2023)
*A. bisporus*	2.24	0.49	6.79	2.5	12.1	34	(Yu et al. 2023)
*Agaricus arvensis*	22.63	0.22	57.12	2.12	0.96	37	(Sileshi et al. 2023)
*Agaricus campestris*	18.3	0.12	43.45	1.17	0.87	—	(Sileshi et al. 2023)
*Amanita caesarea*	19.73	0.21	42.16	1.18	0.42	—	(Niazi and Ghafoor 2021)
*Conocybe tenera*	86.61	0.25	37.10	0.91	0.26	—	(Niazi and Ghafoor 2021)
*Gymnopilus junonius*	14.23	0.23	49.29	1.23	0.48	—	(Niazi and Ghafoor 2021)
*Schizophyllum commune*	15.9	2.0	68.0	—	8.0	399	(Sharma and Gautam 2015)
*Amanita loosei*	15.0	8.2	61.7	14.9	3.4	—	(Choudhary et al. 2015; Sharma and Gautam 2015)
*Clitocybe odora*	41.1	14.3	11.5	12.7	17.4	—	(Choudhary et al. 2015)
*Lactarius phlebophylum*	15.0	9.0	—	64.0	15.2	—	(Reis et al. 2012)
*Polyporus tenuiculus*	16.9	3.2	51.5	9.5	11.6	—	(Phan et al. 2012)
*Termitomyces clypeatus*	18.0	3.8	49.4	7.7	11.2	—	(Phan et al. 2012)

Abbreviations: A, Ash; Carb, Carbohydrate; E, Energy (Kcal/kg); F, Fat; F, Fiber; P, Protein.

A study found that *Coprinus atramentarius* contains 24% carbohydrates, with mannitol being the predominant free sugar. Fresh mushrooms contain 0.91% hemicellulose, 0.28% reducing sugar, 0.59% glycogen, and 0.9% mannitol. *Agaricus bisporus*, also known as the white button mushroom, is a rich source of carbohydrates, including raffinose, sucrose, glucose, fructose, and xylose. These carbohydrates contribute to the mushroom's metabolic processes, taste, nutritional profile, and functional properties. Specifically, raffinose promotes gut health, sucrose provides sweetness, glucose and fructose are essential for cellular energy production, and xylose supports cell wall structure. Furthermore, *A. bisporus* is rich in essential nutrients such as proteins, dietary fiber, vitamins, and minerals, making it a valuable ingredient in functional foods and health‐promoting diets (Kakon et al. 2012).

Mushroom polysaccharides, particularly β‐glucans, enhance immune function by stimulating macrophages, NK cells, and T cells, which aid in pathogen identification, infected cell elimination, and adaptive immune response coordination. They also promote cytokine production, strengthening overall immunity (Sun et al. 2018). *Pleurotus sajor‐caju* powder, with a high carbohydrate content of 60.47 g per 100 g and an energy value of 451.60 cal per gram, contains both digestible and nondigestible carbohydrates, such as mannitol, trehalose, glycogen, and glucose, contributing to its nutritional benefits (Salehi 2019). Additionally, mushroom‐derived polysaccharides, including lentinan, PSP, schizophyllan, and kerstin, are widely studied for their role in immune modulation, autoimmune disease protection, and cancer immunotherapy. Their immune‐modulatory properties depend on structural factors like branching patterns, side‐chain modifications, molecular weight, and receptor interactions, such as dectin‐1. Other well‐researched polysaccharides, including glucans, mannans, galactans, and fructans, provide antioxidant, anti‐inflammatory, gut microbiota‐supporting, and metabolic benefits. Given their extensive applications in immunotherapy, cardiovascular health, neuroprotection, and functional foods, mushroom‐derived polysaccharides hold significant value in both nutritional and pharmaceutical formulations (Zade et al. 2025).

#### 3.1.2 Mushrooms as Sources of Proteins

They are a rich source of protein, containing all essential amino acids, with a protein content varying depending on the species and growth stage, typically ranging from 18% to 37% (Mishra et al. 2015; Samsudin and Abdullah 2019) (Table 1). They, also known as “white veggies” or boneless vegetarian meat, are a rich source of protein, containing 20%–35% more than fruits and vegetables in dry weight. Mushrooms are a rich source of essential amino acids, including lysine and tryptophan, crucial for various physiological functions. As a high‐protein food, second only to animal products, they effectively support muscle growth, tissue repair, and overall health, offering a versatile and nutritious alternative to traditional animal protein. Lysine contributes to protein synthesis, enzyme production, calcium absorption, collagen and elastin formation, immune function, and hormone regulation, while tryptophan, a serotonin precursor, aids in mood, sleep, and appetite regulation, potentially reducing anxiety and depression and promoting relaxation and balanced energy metabolism (Castellanos‐Reyes et al. 2021; Dawadi et al. 2022).

Their protein content is influenced by several factors, including substratum composition, pileus size, harvest timing, and mushroom species. The composition of the substrate—such as sawdust and straw—affects nutrient availability and protein levels. Additionally, the size of the pileus can impact protein concentration. Harvest timing is critical, as they harvested at their peak typically contain higher protein levels. Understanding these variables is essential for maximizing protein yield and enhancing the nutritional value of mushrooms (Li et al. 2022).

*A. bisporus* mycelium, commonly known as the button mushroom, has a high protein content ranging from 32% to 42% by dry weight, making it a nutritious source for food and feed applications. Its high nutritional value makes it an attractive option for vegetarian and vegan diets, while its ability to utilize agricultural by‐products as substrates contributes to a circular economy (Ayimbila and Keawsompong 2023; Waktola and Temesgen 2018). A study found that various mushroom species, including *Agaricus campestris*, *Agaricus arvensis*, *Morchella esculenta*, and *Morchella deliciosa*, have significant protein levels of 30.16%, 28.16%, 34.7%, and 29.16% in their dried mycelium, respectively (Hrudayanath and Sameer 2014).

Although they may lose some protein during storage, they generally have higher protein content than most vegetables (Ayimbila and Keawsompong 2023). A study found that dried mycelium of *A. campestris* , *A. delicious*, *A. arvensis* , and *M. esculenta* has protein levels of 29.16%, 28.16%, 34.7%, and 30.16%, respectively, with higher crude protein content than milk but lower than animal meat (Dawadi et al. 2022). A study discovered that different strains of *Pleurotus*, including eight commercial strains of *P. ostreatus*, three strains of *P. eryngii* , and *P. pulmonarius*, have dry weights of 19.9–31.2, 22.9–23.2, and 30.5 g/100 g, respectively (Raman et al. 2021). Other studies also show that *Pleurotus* strains on commercial substrate have protein contents ranging from 26.7 to 28.2 g/100 g dry weight, while commercial *P. ostreatus* has a protein content of 20.8 g/100 g dry weight (Effiong et al. 2024).

Research outputs indicate that they meet FAO standards for protein quality and contain all the essential amino acids required by adults (Ayimbila and Keawsompong 2023). A study suggests that vegetarians can replace animal protein intake with mushroom protein, which contains 30–50 g/100 g of essential amino acids on a dry matter basis (Effiong et al. 2024). They have varying amounts of essential amino acids, with glutamic acid, L‐aspartic acid, arginine, and threonine present in high concentrations in their protein. Additionally, rare amino acids like γ‐aminobutyric acid and ornithine, and limited amino acids like leucine and lysine, are found in some mushroom varieties (Ayimbila and Keawsompong 2023; Christea et al. 2020).

#### 3.1.3 Mushrooms as Sources of Fats

They are a rich source of essential fatty acids, including omega‐3 and omega‐6, essential for overall health. These fatty acids, like linoleic acid, are anti‐inflammatory, aid in brain function, heart health, and reduce chronic disease risk. They promote skin health, immune function, and metabolism regulation. Linolenic acid is crucial for cell membrane integrity and cardiovascular health. Despite their low fat content—accounting for just 2%–6% of their dried bulk compared to carbohydrates and proteins—they are abundant in unsaturated fatty acids, including monounsaturated and polyunsaturated fats, which are essential for heart health and general well‐being (Li et al. 2016) (Table 1).

Monounsaturated fatty acids (MUFAs), such as oleic acid, help reduce bad cholesterol levels, thereby lowering the risk of heart disease and stroke. Polyunsaturated fatty acids (PUFAs), including omega‐3 and omega‐6, are vital for normal body functions, supporting brain and heart health. Omega‐3 s, such as alpha‐linolenic acid (ALA), eicosapentaenoic acid (EPA), and docosahexaenoic acid (DHA), have anti‐inflammatory properties, while omega‐6 s are important for skin health, hormone regulation, and immune system support (Cateni et al. 2022).

However, the fat content varies between species. For example, *Suillus granulatus* has a fat content of 2.04%, *Suillus luteus* contains 3.66%, and *Agaricus campestris* has 2.32% (Bengu 2020; Łysakowska and Sobota 2023). A study found that *A. bisporus* has a total fat content ranging from 1.66 to 2.2 g per 100 g (Dawadi et al. 2022). Research indicates that they are primarily rich in unsaturated fatty acids, such as oleic, linoleic, and linolenic acids, along with polyunsaturated fatty acids, with levels ranging from 0.0% to 81.1% of total fatty acids per 100 g (Brugnari et al. 2016; Sande et al. 2019). Studies also show that mushrooms are rich in unsaturated fatty acids like oleic and linoleic acids, as well as saturated fatty acids like palmitic acid, with a 2:1 ratio of unsaturated to saturated fatty acids (Brugnari et al. 2016). Linolenic acid is a key precursor to 1‐octen‐3‐ol, a significant fragrant component in mushrooms that contributes to their distinctive flavor (Tan et al. 2024).

#### 3.1.4 Mushrooms as Sources of Fibers

They are a rich source of high‐quality fiber, comprising both soluble and insoluble fibers. Soluble fibers, such as beta‐glucans and pectin, help reduce cholesterol levels by binding to cholesterol molecules in the digestive tract (Flores et al. 2024). When consumed, these fibers form a gel‐like substance that traps cholesterol and bile acids, preventing their absorption into the bloodstream. This process lowers total cholesterol and low‐density lipoprotein (LDL) cholesterol, thereby reducing the risk of heart disease and stroke. Consuming foods rich in soluble fiber can promote cardiovascular health and overall well‐being (Suresh et al. 2024).

In addition to soluble fibers, they are also rich in insoluble fibers, including chitin, lignin, cellulose, and xyloglucans. Chitin provides structural support and adds bulk to the stool, while lignin contributes to cell wall integrity and enhances stool bulk. Cellulose adds rigidity and strength to the mushroom's structure, while beta‐glucans support digestive health, aid in cholesterol reduction, and bolster immune function. Xyloglucans, which are polysaccharides found in some mushrooms, also contribute to the fiber content and structural integrity (Flores et al. 2024).

Mushrooms contain insoluble fibers that aid in maintaining digestive health by preventing constipation, aiding weight management, and facilitating food and waste movement. Regular consumption reduces the risk of digestive disorders. Incorporating mushrooms into a balanced diet improves cardiovascular health, digestive function, and overall well‐being (SSingh et al. 2025). Mushrooms are rich in insoluble fiber, comprising 20%–33%, and soluble fiber, with free sugars accounting for only 11% of the total. The insoluble fiber is primarily chitin and β‐glucan, which make up 80%–90% of their dry weight (Marçal et al. 2021; Naeem et al. 2020; Siroha et al. 2021; Zhou et al. 2024).

#### 3.1.5 Mushrooms as Sources of Vitamins

They are considered the best sources of essential vitamins, particularly group B vitamins like thiamine, riboflavin, pyridoxine, pantothenic acid, nicotinamide, folic acid, and cobalamine, along with other vitamins like ergosterol, biotin, tocopherols, and vitamin D2 (KKumar et al. 2021) (Table 2). They are a rich plant‐based source of niacin, providing over 25% of an adult's daily requirement in just 100 g. They also contain vitamin B12, which is often insufficient in vegetarian diets due to their reliance on animal sources. With a B12 content of 0.32–0.65 mg/g, 3 g of fresh mushrooms are a good vegetarian source (Heleno et al. 2012).

**TABLE 2:** Vitamin contents of wild edible mushrooms (mg/100 g) on a dry mass basis.

Mushroom species	Th	R	V.D	NA	AA	FA	References
*Pleurotus citrinopileatus*	0.16	0.94	0.4	22.2	0.9	97.0	(Sileshi et al. 2023)
*Polyporus tenuiculus*	0.94	0.09	0.2	6.95	18.1	158.5	(Niazi and Ghafoor 2021)
*Termitomyces microcarpus*	0.17	0.06	—	3.46	13.6	120	(Niazi and Ghafoor 2021)
*Termitomyces tyleranus*	0.35	0.07	—	7.31	14.4	70	(Sileshi et al. 2023)
*Termitomyces clypeatus*	0.19	0.05	—	7.3	15.1	150	(Paloi et al. 2023)
*Volvariella speciosa*	0.24	0.06	0.6	2.66	11.1	159	(Raman et al. 2021)
*Volvariella volvacea*	0.14		0.1	2.3	—	147	(Musieba et al. 2013)
*Agaricus biosporus*	0.025		0.3	3.8	—	173	(Nakalembe et al. 2015)
*Flamulina velitpes*	0.03		0.1	2.4	—	—	(Nakalembe et al. 2015)
*Lentilula edodes*	0.024		0.4	2.0	13.5	—	(Sileshi et al. 2023)
*Pleurotus ostreatus*	0.031		0.7	—	21.2	154	(Raman et al. 2021)

Abbreviations: AA, ascorbic acid; FA, folic acid; NA, nicotinic acid; R, riboflavin; Th, thiamine; V.D, vitamin D.

Cultivated mushrooms are a rich source of essential nutrients, including provitamin A, which supports vision, immune function, and cell growth; fat‐soluble vitamins K and E, which are crucial for blood clotting and bone health; and vitamin C, which supports the immune system, promotes collagen production, and enhances iron absorption. Although their concentrations may be lower than other sources, incorporating cultivated mushrooms into a balanced diet can still provide nutritional benefits and maintain overall health (Kumar et al. 2021). A previous study found that certain *A. bisporus* species have riboflavin levels similar to those found in eggs and cheese, while *P. eryngii* , *P. ostreatus*, and *A. bisporus* have higher niacin levels, with *P. eryngii* having the highest amount (5.9 mg/kg) (Zhou et al. 2024).

Wild mushrooms have higher concentrations of vitamin D compared to cultivated varieties due to their growth conditions and exposure to sunlight, which converts ergosterol into vitamin D2. Cultivated mushrooms, grown in controlled environments, experience reduced vitamin D synthesis. Although some commercial producers use UV light treatment to enhance their vitamin D levels, cultivated mushrooms generally have lower concentrations than wild varieties (Rondanelli et al. 2023). In contrast, cultivated mushrooms, grown in controlled environments, typically have lower vitamin D levels but are rich in ergosterol, a precursor to vitamin D2 (Cateni et al. 2022).

#### 3.1.6 Mushrooms as Sources of Minerals

They are rich in essential minerals like K, P, Na, Ca, Mg, and trace elements like Cu, Zn, Fe, Mo, and Cd, which are crucial for human health (Raman et al. 2021) (Table 3). A study found that potassium alone accounts for 45% of mushroom ash, while K, P, Na, and Mg contribute 56%–70% of the total ash (Dawadi et al. 2022). Wild edible mushrooms have higher mineral content than cultivated varieties due to factors like natural growth conditions, soil composition, symbiotic relationships, growth stimulation, and species diversity. They grow in diverse environments, accessing a broader range of minerals from soil and organic matter. Soil composition influences mineral content, while competition from other organisms and environmental stressors stimulates growth and mineral absorption mechanisms (Gałgowska and Pietrzak‐fie 2020). *A. bisporus*, a common cultivated mushroom, is rich in essential minerals such as potassium, phosphorus, magnesium, zinc, iron, selenium, and copper (Joshi et al. 2021). These minerals are crucial for human health, helping to maintain electrolyte balance, nerve function, muscle contractions, cardiovascular health, reducing inflammation, supporting immune function, promoting wound healing and cognitive health, aiding in oxygen transport and red blood cell formation, and contributing to immune defense. They also play a role in red blood cell production, brain function, and iron metabolism. Consequently, *A. bisporus* mushrooms are a valuable component of a healthy diet, enhancing immunity, heart health, and metabolic function, making them a beneficial alternative to traditional nutrient sources (Ayimbila and Keawsompong 2023).

**TABLE 3:** Mineral content of commonly cultivated mushrooms, measured in mg/100 g on a dry mass basis.

Mushroom species	Mineral content (mg/100 g)										
	Na	K	P	Ca	Mg	Fe	Zn	Mn	Se	Cu	References
*Volvariella volvacea*	7	309	89	8	8	0.14	—	0.05	2.1	1.56	(Sileshi et al. 2023)
*A. bisporus*	11	364	108	3	9	0.31	1.2	0.067	18.6	2.3	(Sileshi et al. 2023)
*Flammulina velutipes*	8	359	105	—	16	1.15	1.28	0.075	2.2	2.01	(Sileshi et al. 2023)
*Lentinula edodes*	16	304	112	2	20	0.41	—	0.23	5.7	1.9	(Niazi and Ghafoor 2021)
*Pleurotus ostreatus*	10	420	120	3	18	1.33	1.91	0.113	2.6	—	(Niazi and Ghafoor 2021)
*Morchella esculenta*	6	342	3.31	7	8.2	1.21	—	0.28	1.9	7.8	(Sharma and Gautam 2015)
*Amanita loosii*	10.0	407	—	27	105	1.6	1.01	0.02	2.3	6.2	(Choudhary et al. 2015)
*Auricularia auricular*	20.0	10.6	13.9	5.5	1.09	1.2	—	0.09	—	—	(Sharma and Gautam 2015)
*Auricularia polytricha*	10.9	294	623	88.6	83.5	1.03	1.51	—	2.24	3.4	(Sharma and Gautam 2015)
*Cantharellus contortus*	7.0	370	—	8.0	106	75.2	1.7	—	—	—	(Sileshi et al. 2023)
*Laetiporus sulphureus*	4.2	433.3	542	13.3	13.9	8.7	2.66	0.04	3.2	3.6	(Obodai et al. 2014)
*Lentinus squarrosulus*	5.0	114.6	543	4.7	1.42	6.10	1.2	—	—	3.72	(Obodai et al. 2014)
*P. citrinopileatus*	3.65	15.4	4.44	1.57	1.48	5.60	1.08	—	2.2	2.8	(Sileshi et al. 2023)
*Coprinopsis cinerea*	0.33	52.1	0.42	0.52	46.2	53.5	6.83	6.79	—	23.9	(Musieba et al. 2013)
*Cantharellus cibarius*	0.29	47.9	0.58	0.42	46.2	6.79	11.1	7.68	—	4.36	(Musieba et al. 2013)
*Russula brevispora*	0.31	35.5	0.51	0.53	217	7.17	6.76	11.4	—	7.17	(Nakalembe et al. 2015)
*Russula integra*	1.27	41.0	0.24	1.27	327	56.2	10.5	7.28	—	3.33	(Nakalembe et al. 2015)

Mushrooms grown in industrial areas can absorb heavy metals like cadmium, lead, arsenic, copper, nickel, silver, chromium, and mercury. Factors affecting mineral content include species, age, fruiting body diameter, and substrate type. Without proper management, mushrooms can accumulate harmful metals (Bucurica et al. 2024). Studies have identified elevated levels of nickel, chromium, and cadmium in mushrooms harvested from industrial areas, reflecting the presence of environmental contaminants. Among agricultural crops, edible mushrooms are notable for their high concentrations of heavy metals, highlighting their ability to absorb toxic metals from the soil (Effiong et al. 2024; Kaleem et al. 2023). Mushroom fruiting bodies can accumulate toxic metals and metalloids in polluted environments, posing health risks. Therefore, cultivation should occur in low‐pollution areas (Maryam et al. 2022).

### 3.2 Mushrooms for Animals Forage

Agricultural crop residue is crucial for animal feed, boosting milk production and body mass, but its nutritional quality is often below average, necessitating the use of enriched nutrient sources (Bellere et al. 2023). Spent mushroom substrate feeds offer nutritional and economic benefits by reducing bovine feed input costs, making them cheaper than standard feeds. However, pelleting and transportation costs may offset these benefits, requiring further research (Van Wyk 2022).

Oyster mushroom spent substrate (OMSS) is a nutrient‐dense animal feed supplement, rich in proteins, microorganisms, and essential nutrients. It contains extracellular enzymes for digestion and nutrient absorption in animals (Mthana and Mthiyane 2024). The substrate is also packed with macronutrients like nitrogen, phosphorus, and potassium, polysaccharides such as beta‐glucans, which boost immune function, and various vitamins, particularly B‐vitamins, contributing to overall animal health. OMSS, a sustainable and cost‐effective alternative to traditional feed, promotes gut health and is suitable for livestock and poultry due to its nutritional value (Martín et al. 2023). Mushrooms, rich in antioxidants and antibacterial properties, can enhance animal and poultry health by acting as probiotics in poultry feed, improve pig body weight, and increase fish production with reduced feed costs (Adanikin and Kayode 2019).

Agricultural by‐products, including crop residues and grains, are rich in essential nutrients but difficult for cattle to digest due to their high cellulose, hemicellulose, and lignin content. This leads to lower feed utilization rates, slower growth, and reduced productivity, necessitating the need for feed supplements or processing techniques (Hafifah et al. 2018). The European Union is reducing its 70% reliance on imported soy‐based animal feed, which has environmental and logistical implications. The high demand has led to deforestation and compromised nutritional value. To address these issues, the EU is exploring sustainable alternatives like spent mushroom substrates (SMS), which are nutrient‐dense, protein‐rich, and economically viable, making them easier to digest than traditional feed. This approach promotes circular economy practices, reduces dependency on imported soy, supports local farmers, and creates a more resilient and sustainable animal feed system. This shift towards locally sourced feed could improve food security, reduce environmental impact, and ensure a steady supply of high‐quality feed within the EU (Grimm and Wösten 2018).

Traditional cattle feeds, including straw and agricultural residues, lack energy, protein, and mineral content due to the high digestibility of hard‐to‐digest cell wall components like cellulose, hemicellulose, and lignin (Chugh et al. 2022). Chemical treatment with sodium hydroxide or ammonia can enhance nutritional value, but these methods are economically and environmentally unfavorable (Bhandari et al. 2023; Shah et al. 2023). Delignification, a biological process involving the decomposition of cell wall components, is a promising alternative to traditional methods. Fungi, particularly mushrooms, are efficient decomposers of agricultural residues and lignocellulosic biomass, which can be converted into valuable food sources for humans and cattle (Blasi et al. 2023). A study found that incorporating mushroom waste from *A. bisporus* production into ruminant feed, ensiled with hay and corn, increased crude protein, calcium, and acid detergent fiber levels (Nidadavolu et al. 2012).

### 3.3 Application of Mushrooms in Medicine

Mushrooms serve as a valuable source of functional foods with therapeutic properties that support disease prevention and management. Beyond basic nutrition, they provide essential vitamins, minerals, antioxidants, fiber, and probiotics, contributing to overall health. These functional foods can occur naturally or be enriched with additional nutrients. For centuries, various cultures have utilized mushrooms to promote well‐being, recognizing their immunomodulatory and antineoplastic benefits in maintaining health and preventing illnesses (Das et al. 2021). Recently, there has been a rapid increase in interest in the pharmaceutical potential of mushrooms, with some suggesting that they are mini‐pharmaceutical factories producing compounds with remarkable biological properties (Niego et al. 2021).

Advancements in cancer research have paved the way for new medications designed to target abnormal molecular and biochemical signals involved in disease progression. As functional foods, mushrooms possess a rich array of bioactive compounds, including polysaccharides, proteins, lipids, and secondary metabolites such as terpenoids, phenolics, and alkaloids, which contribute to their therapeutic potential (Pathak et al. 2022). These compounds have the potential to modulate biological activities, including anti‐inflammatory, antioxidant, and immunomodulatory effects. Polysaccharides in mushrooms enhance the immune response and may inhibit tumor growth. Terpenoids and phenolic compounds can disrupt cancer cell signaling pathways, inhibiting proliferation and inducing apoptosis (Bains et al. 2021).

Integrating cancer research with mycology holds promise for innovative cancer treatments using mushroom‐derived bioactive compounds. These compounds offer antioxidant and antitumor effects, while also providing antibacterial and anti‐allergic benefits for overall health (Nandi et al. 2024). Their immunomodulating effects help regulate the immune system, strengthening its ability to combat infections and illnesses. Some mushroom‐derived compounds offer cardiovascular protection, detoxifying toxins, and being effective against infections. They also contribute to improved metabolic and systemic health, enhancing immune function, reducing inflammation, providing hepatoprotection, lowering lipid levels, preventing excessive blood clotting, regulating blood sugar levels, and maintaining healthy blood pressure. Further exploration could lead to novel dietary supplements, functional foods, and pharmaceutical applications (Chugh et al. 2022).

Mushrooms are a valuable functional food due to their bioactive compounds, which can help combat obesity and cognitive decline. Their high fiber content promotes satiety, while beta‐glucans enhance gut health and lower cholesterol. Mushrooms also contain neuroprotective compounds, which help preserve brain function. Their essential B vitamins support energy metabolism and neurotransmitter activity. Incorporating mushrooms into a balanced diet improves digestive function, cardiovascular health, and cognitive performance (Das et al. 2022; Echer et al. 2022).

Mushrooms and other fungi exhibit over 100 medicinal properties, including antioxidant, anticancer, antidiabetic, anti‐allergic, immunomodulating, cardiovascular protective, anticholesterolemic, antiviral, antibacterial, antiparasitic, antifungal, detoxification, and hepatoprotective effects, which help guard against tumor development and inflammatory processes (Bhambri et al. 2022). Research shows that regular consumption of mushrooms or their products can effectively prevent and treat various ailments. Edible mushrooms and their active components have demonstrated beneficial effects on hyperglycemia and hypercholesterolemia (Shamim et al. 2023). They are abundant in acidic polysaccharides, dietary fiber, and antioxidants, including vitamins C, B12, and D, folate, ergothioneine, and polyphenols, which may also contribute to their anti‐inflammatory, hypoglycemic, and hypocholesterolemic effects (Vinjusha and Tk 2021) (Table 4).

**TABLE 4:** Bioactive compounds isolated from mushrooms and their medical applications.

Mushroom species	Bioactive compounds	Medical applications (Bioactivity)	References
*A. bisporus*	Pyrogallol hydroxybenzoic acid derivatives, Flavonoids	Anti‐inflammatory	(Moro et al. 2012)
*Agaricus macrosporus*	Agaricoglycerides	Anti‐inflammatory	(Han and Cui 2012)
*Volvariella volvacea*	Glycoproteins	Enhance insulin secretion, antiaging property.	(Naeem et al. 2020)
*Trametes versicolor*	Polysaccharide‐K (Krestin)	Antitumor activities, lowers cholesterol	(Naeem et al. 2020)
*Pleurotus sajor‐caju*	Lovastatin polysaccharide	Antidepressant activity	(Naeem et al. 2020)
*Lentinula edodes*	Eritadenine, Lentinan	Antioxidant, anticancer, antiaging	(Naeem et al. 2020)
*Grifola frondosa*	Grifloan, Lectins	Antibiotic properties, anticancerous properties.	(Naeem et al. 2020)
*Agaricus subrufescens*	Glycoprotein, b‐(1, 3)‐glucan, with b‐(1,6)‐glucan branch	Immunomodulatory	(Flores et al. 2024)
*Agaricus brasiliensis*	Protein and polysaccharides fractions	Immunomodulatory	(Smiderle et al. 2011)
*Agaricus rufotegulis*	Polysaccharides fractions	Immunomodulatory	(Rathee et al. 2012)
*Agrocybe cylindracea*	b‐Glucans	Anti‐oxidant	(Zhang et al. 2024)
*Albatrellus ovinus*	Grifolin and grifolin derivatives	Anti‐inflammatory, anti‐oxidant	(Hettwer et al. 2017)
*Albatrellus caeruleoporus*	Phenolic compound	Anti‐inflammatory	(Santos and Magalhães 2014)
*Albatrellus caeruleoporus*	Grifolinones A, B	Anti‐inflammatory	(Zhang et al. 2023)
*Antrodia camphorata*	Glycoprotein ACA	Immunomodulatory	(Yang et al. 2022)
*Taiwanofungus camphoratus*	Diterpenes	Neuroprotective	(Wang et al. 2019)
*G. lucidum*	Polysaccharides, triterpenoids	Increases insulin secretion, decrease blood glucose, improves ovulation	(Naeem et al. 2020)
*Flammulina velutipes*	Polysaccharide, flammulina polysaccharide protein, peptide glycans	Lower cholesterol, anticancer agent	(Naeem et al. 2020)
*Cordyceps sinensis*	Cordycepin	Lower cholesterol, prevents cardiovascular disorders.	(Naeem et al. 2020)
*Auricularia auricula*	Acidic Polysaccharides	Decrease immune system depression, prevents cancer	(Naeem et al. 2020)
*Cordyceps militaris*	Cordycepin	Antioxidant; antihyperlipidaemic; hepatoprotective	(Paisansak et al. 2021)
*Lentinula edodes*	Peptides	ACE inhibitory	(Wongaem et al. 2021)
*Schizophyllum commune*	Peptides	Anti‐oxidant	(Li et al. 2023)
*Hericium erinaceus*	Peptides	Anti‐oxidant	(Song et al. 2020)
*Albatrellus confluens*	Grifolin	Osteosarcoma	(Ma et al. 2018)
*Auricularia auricular*	Polysaccharide	Liver cancer HepG2	(Joseph et al. 2018)
*Amauroderma rude*	Ergosterol	Breast cancer	(Joseph et al. 2018)
*Antrodia camphorata*	Polysaccharide	Hepatocellular carcinoma	(Chang et al. 2011)
*Agaricus. camphorata*	Acetylantroquinonol B	Colorectal cancer	(Tomasz 2023)
*Coprinus comatus*	Laccases	Antitumor and antiviral	(Zhao et al. 2014)
*Cerrena unicolor*	Laccases	Antitumor (leukemic cell)	(Matuszewska et al. 2016)
*Coriolus versicolor*	PSK, Krestin (Protein bound polysaccharide)	Immunostimulant property and inhibit tumor growth	(Joseph et al. 2018)
*Flammulina velutipes*	Ribosome‐inactivating proteins	Antiviral (HIV‐1)	(Ma et al. 2018)
*Fomes fomentarius*	Polysaccharide (MFKFAP1β)	Lung cancer	(Joseph et al. 2018)
*Ganoderma applanatum*	Lectin	Antitumor (HT‐29 colonadenocarcinoma cells)	(Ma et al. 2018)
*Hypsizigus marmoreus*	Fungal immunomodulatory proteins, lectine	Antiproliferative (Human leukemia) and hepatoma cells	(Ma et al. 2018)
*Lentinus crinits*	Polysaccharide	Panepoxydone (PP)	(Joseph et al. 2018)
*Lyophyllum shimeiji*	Ribonucleas	Antitumor	(Zhao et al. 2014)
*Macrolepiota procera*	Lectin	Antitumor activity	(Ma et al. 2018)
*Naematoloma fasciculare*	Triterpenoids	Antiproliferative activities	(Joseph et al. 2018)
*Phellinus linteus*	Protein‐bound, polysaccharide	Antitumor (Colon cancer)	(Joseph et al. 2018)
*P. eryngii*	Hetero polysaccharide	Antitumor	(Ma et al. 2018)
*P. ostreatus*	Homogeneous polysaccharide	Antitumor	(Ma et al. 2018)
*P. eryngii*	Homogeneous polysaccharide	Antitumor	(Ma et al. 2018)
*P. ostreatus*	Lectin	Immuno‐modulatory	(Ma et al. 2018)
*P. cornucopiae*	Monoterpenoids	Inhibitory activity	(Wang et al. 2014)
*P. ferulae*	Terpenoids	Melanoma/Gastric cancer	(Joseph et al. 2018)
*P. pulmonarius*	Protein‐bound polysaccharide	Antitumor (Liver cancer)	(Xu et al. 2012)
*Ramaria formosa*	Ribonucleas	Antiviral activity Inhibitory activity (HIV‐1)	
*Schizophyllum commune*	Sonifilan (Polysaccharide SPG)	Immunostimulant	(Joseph et al. 2018)
*Stachybotrys chlorohalonata*	Fungal immunomodulatory proteins	Immuno‐modulatory	(Ma et al. 2018)
*Termitomyces clypeatus*	Sugar entities	Antitumor activity (ovary, breast, and Brain)	(Joseph et al. 2018)
*Lepista personata*	Ribonuclease	Anti‐HIV viral	(Zhang et al. 2015)
*Pleurotus ostreatus*	Ostreatin	Antitumor	(Abdullah et al. 2022)
*Auricularia auricula‐judae*	Lectin	Antimicrobial	(Nnamdi et al. 2020; Wang et al. 2014)
*Pleurotus geesteranus*	Protein hydrolysates	Neuroprotective effects	(Wu et al. 2021)
*Inonotus obliquus*	Poliphenols (phenolic acids, flavonoids, quinones, and styrylpyrones)	Anti‐inflammatory hypoglycemic effect, and Antiglication effect	(Peng and Shahidi 2022)
*Ophiocordyceps sinensis*	Cordycepic acid	Anti‐inflammatory, antioxidant, antiaging	(Yarnell and Ahg 2007)
*Auricularia auricula*	Glucan	Hyperglycemia, Antitumor and immunomodulatory	(Santos and Magalhães 2014)
*Boletus edulis*	Polysaccharides	Anti‐inflammatory	(Moro et al. 2012)
*Cantharellus cibarius*	Pyrogallol	Anti‐inflammatory	(Moro et al. 2012)
*Calvatia gigantea*	Calvacin	Antitumor	(Rathee et al. 2012)
*Caripia montagnei*	Polysaccharides (glucans)	Anti‐inflammatory	(Santos and Magalhães 2014)
*Clitocybe maxima*	Laccase	Antitumor	(Charles Guest and Rashid 2016)
*Cortinarius infractus*	6‐hydroxyinfractine, infractopicrine	Anti‐neurodegenerative	(Abitbol et al. 2022)
*Craterellus cornucopioides*	Myricetin	Anti‐oxidant	(Palacios et al. 2011)
*Craterellus tubaeformis*	Polysaccharides	Anti‐inflammatory	(Yakobi et al. 2023)
*Cyathus africanus*	Diterpenoid (neosarcodonin, cyathatriol, and 11‐O acetylcyathatriol)	Anti‐inflammatory	(Zorrilla and Evidente 2022)
*Daldinia concentrica*	1‐(3,4,5‐trimethoxyphenyl) ethanol, caruilignan C	Neuroprotective	(Lee et al. 2002)
*Dictyophora indusiata*	Dictyophorine A and B	Anti‐neurodegenerative	(Abitbol et al. 2022)
*Phallus indusiatus*	Dictyoquinazol A, B, and C	Neuroprotective	(Wani et al. 2015)
*Elaphomyces granulatus*	Syringaldehyde	Anti‐inflammatory	(Qian et al. 2012)
*Flammulina velutipes*	Flammulin (protein), Polysaccharides	Antitumor, Anti‐inflammatory	(Kumar et al. 2021)
*Fomitopsis pinicola*	Polysaccharides	Anti‐inflammatory	(Wani et al. 2015)
*Ganoderma microsporum*	Protein GMI	Immunomodulatory	(Teo et al. 2020)
*Ganoderma pfeifferi*	Sesquiterpenoid hydroquinones (lucialdehyde D, ganoderone A, ganoderone C)	Antibacterial, anti‐fungal, anti‐viral	(Galappaththi et al. 2023)
*Geastrum saccatum*	Polysaccharides (b‐glucans)	Anti‐inflammatory	(Fahlquist‐Hagert et al. 2022)
*Ganoderma tsugae*	Fip‐gts (protein)	Immunomodulatory	(Teo et al. 2020)
*Polyporus tsugae*	(1–6‐monoglucosyl‐branched)	Immunomodulatory, antitumor, anti‐viral, hepatoprotective	(Kidd 2016)
*H. erinaceus*	Phenol‐analogous compounds (hericenons C, D, E, F, G, H)	Anti‐oxidant	(Gariboldi et al. 2023)
*Hypsizygus marmoreus*	Ergosterol, manitol	Anti‐oxidant, anti‐inflammatory	(Aditya et al. 2025)
*Hypsizygus tessellatus*	Trehalose, methionine	Anti‐allergic	(Wong et al. 2020)
*Lactarius deliciosus*	Pyrogallol, Flavonoids	Anti‐inflammatory	(Moro et al. 2012)
*L. flavidulus*	Polysaccharides	Anti‐inflammatory	(Kosti et al. 2023)
*Lactarius rufus*	Polysaccharides: (1,3), (1,6) β‐D‐glucans	Anti‐inflammatory	(Caroline et al. 2013)
*Lentinula polychrous*	Catechin	Anti‐oxidant	(Thetsrimuang et al. 2011)
*Lentinula squarrosulus*	Catechin	Anti‐oxidant	(Thetsrimuang et al. 2011)
*Lenzites betulina*	Betulinan A	Anti‐oxidant	(Rathee et al. 2012)
*Lignosus rhinocerus*	Polysaccharides‐protein	Anticancer	(Klaic et al. 2015)
*Lyophyllum decastes*	Polysaccharides:(1, 3) and (1,6) β‐D‐glucans	Anti‐inflammatory	(Vetter 2023)
*Mycoleptodonoides* *aitchisonii*	3‐(hydroxymethyl)‐4‐methylfuran‐2(5H)‐one, (3R,4S,1′R)‐3‐(1′‐hydroxy‐ethyl)‐4 methyldihydrofuran‐2(3H)	Anti‐neurodegenerative	(Choi et al. 2016)
*Morchella esculenta*	Heteroglycan Galactomannan, b‐1,3‐D‐glucan	Hyperglycemia, antitumor	(Wani et al. 2015)
*Phellinus linteus*	Glucans	Antitumor	(Kim and Iwahashi 2015)
*Pholiota adiposa*	Lectin (glycoprotein)	Antitumor, anti‐viral	(Zou and Hou 2017)
*Pholiota nameko*	Polysaccharides	Anti‐inflammatory	(Xie et al. 2019)
*Pleurotus citrinopileatus*	Glycoprotein (PCP‐3A)	Antitumor, anticancer	(Chen et al. 2011)
*Pleurotus florida*	β‐glucans	Anti‐oxidant	(Lavi et al. 2010)
*Pleurotus pulmonarius*	Polysaccharides b (1,3)‐glucopyranosyl	Anti‐inflammatory	(Lavi et al. 2010)
*Psilocybe cubensis*	Psilocybin (psilocin: 4‐hydroxydimethyltryptamine)	Antidepressant (Psychotherapy)	(Carhart‐harris et al. 2012)
*Psilocybe samuiensis*	Psilocybin (psilocin: 4‐hydroxydimethyltryptamine)	Antidepressant (Psychotherapy)	(Kraehenmann et al. 2014)
*Psilocybe Mexicana*	Psilocybin (psilocin: 4‐hydroxydimethyltryptamine)	Antidepressant (Psychotherapy)	(Mckay et al. 2011)
*Russula lepida*	Lectin (glycoprotein)	Antitumor	(Islam et al. 2016)
*Sparassis crispa*	β‐Glucan	Immunomodulatory	(Fahlquist‐Hagert et al. 2022)
*Termitomyces albuminosus*	Termitomycesphins (cerebrosides)	Anti‐ neurodegenerative	(Yuan et al. 2012)
*Tremella aurantiaalba*	Heteroglycan	Immunomodulatory	(Du et al. 2010)
*Tremella mesenterica*	Glucurono‐xylomannan polysaccharide	Hypoglycemic, immunomodulatory	(Liu et al. 2023)
*Tricholoma giganteum*	Trichogin (protein)	Antifungal	(Stuper‐szablewska and Siwulski 2021)
*Tricholoma mongolicum*	Laccase	Anti‐viral, antitumor	(Santos et al. 2015)

*Pleurotus* species have medicinal properties, including anti‐inflammatory, antioxidant, and antitumor properties, used in chronic diseases like hypertension and hypercholesterolemia, radiation treatments, and chemotherapy (Echer et al. 2022). *Pleurotus* species contain bioactive compounds with antibacterial and antifungal properties, providing natural pathogen defense. These compounds inhibit both Gram‐positive and Gram‐negative bacteria and suppress fungal growth by disrupting cell membranes or metabolic processes. This antimicrobial potential offers valuable applications in medicine and agriculture, with extracts potentially serving as sustainable alternatives to synthetic agents for improved food safety and public health (Gashaw et al. 2020).

*Agaricus blazei*, used in general medicine for diseases like cancer, hepatitis, diabetes, and hyperlipidemia, has gained significant interest as a dietary supplement due to its antitumor, anticarcinogenic, antiviral, anti‐inflammatory, hypoglycemic, and antihypertensive properties (Chugh et al. 2022). These mushrooms offer benefits for chronic breast and joint diseases, cardiovascular health (lowering cholesterol, improving circulation), and various conditions like night sweats, tuberculosis, rheumatism, gout, jaundice, dropsy, and intestinal worms. Their antitumor, anti‐viral, and anticancer properties, particularly through immune modulation, also make them effective for hypertension, renal issues, and diabetes. Furthermore, polysaccharide‐protein complexes from mycelial cultures and lectins from edible mushrooms contribute to their medicinal value (Jayachandran et al. 2017).

Research has shown that *Ganoderma lucidum* contains various bioactive compounds that contribute to its therapeutic effects. Among these compounds are polysaccharides, triterpenoids, and peptides, which are believed to play a vital role in improving metabolic functions and supporting immune health. These properties are crucial for individuals battling chronic illnesses such as HIV/AIDS, as they may help bolster the immune system and combat the virus's effects on the body (Seweryn and Gamian 2021).

These containing psychoactive compounds have been utilized for centuries across various cultures, particularly polypore species such as *G. lucidum* (commonly known as Reishi). These polypore mushrooms, especially Reishi, have been valued not only for their physical health benefits but also for their psychological and spiritual properties. They are now recognized in modern science for their multifaceted healing effects. Research is actively exploring the therapeutic benefits of these mushrooms, highlighting their potential as natural remedies for various health issues. By delving into their psychoactive effects and holistic healing potential, individuals can enhance their mental and physical health, reconnect with themselves, and foster a deeper understanding of their overall well‐being (Łysakowska and Sobota 2023).

Extracts from the fruiting bodies and mycelia of *Ganoderma* and *Volvariella* species exhibit significant in vitro antioxidant and antimutagenic properties, suggesting their potential in preventing and managing a range of diseases, including cancer. Their potent antioxidant activity helps neutralize damaging free radicals, unstable molecules that can harm cellular components like DNA, lipids, and proteins, contributing to degenerative conditions such as cardiovascular and neurodegenerative diseases (Sułkowska‐ziaja et al. 2022). Moreover, these extracts demonstrate antimutagenic properties, effectively protecting cellular DNA from mutations induced by environmental toxins and other harmful agents, thereby hindering the development of diseases linked to genetic instability. This highlights the potential of *Ganoderma* and *Volvariella* extracts as preventative and therapeutic agents, emphasizing the need for further in vivo and clinical studies to fully assess their efficacy and safety in humans (Oke et al. 2022).

The extract from *Pleurotus cornucopiae* has been found to have potent antigenotoxic and bio‐antimutagenic properties. It effectively reduced genetic damage caused by mutagens on bacterial strains like *Salmonella typhimurium* and *Escherichia coli* , demonstrating its potential protective effects against DNA damage. The extract's antigenotoxic properties can prevent or reverse the harmful effects of toxic substances that can lead to mutations. It also counteracts mutagenic effects induced by environmental pollutants or chemical agents, which increase the risk of mutations and cancer in living organisms. The bio‐antimutagenic effects suggest it can inhibit the mutagenic activity of certain compounds, protecting the integrity of genetic material (Anusiya et al. 2021). These findings highlight the potential of *P. cornucopiae* as a culinary ingredient and functional food with health benefits, particularly in cancer prevention and genetic protection. Further research could lead to the development of natural therapeutic agents that enhance health and reduce the risk of mutation‐related diseases (Martinez‐Medina et al. 2021).

*P. citrinopileatus*, commonly known as the golden oyster mushroom, exhibits potent antihyperlipidemic effects, aiding in the reduction of elevated blood lipid levels, particularly cholesterol and triglycerides—key contributors to cardiovascular disease. Its bioactive compounds regulate lipid metabolism by limiting absorption, promoting excretion, and enhancing utilization (Sheng et al. 2019). Regular consumption of *P. citrinopileatus* extracts may help decrease harmful cholesterol while potentially boosting beneficial cholesterol. Packed with polysaccharides, vitamins, and minerals, this mushroom supports overall metabolic health. Additionally, its rich antioxidant profile helps combat oxidative stress, a factor in dyslipidemia and other metabolic disorders (Sharma et al. 2024).

#### 3.3.1 Antimicrobial Activity

Studies have shown that both edible and nonedible mushrooms possess antimicrobial properties and bioactive compounds, effectively combating pathogenic microorganisms (Alves et al. 2012; Gebreyohannes et al. 2019b). *Lentinula edodes*, *Boletus*, *Ganoderma*, and *Lepista* species are effective against both Gram‐positive and Gram‐negative bacteria. The antibacterial activity of mushrooms primarily derives from their phenolic compounds, which disrupt cell membranes. However, this antibacterial activity is influenced by factors such as the variety of mushroom, phenolic content, and the type of pathogenic bacteria (Gebreyohannes et al. 2019a; Gebreyohannes and Sbhatu 2023). A study found that certain mushrooms contain phenolic compounds with significant antibacterial properties. *Lactarius deliciosus* extract showed antibacterial activity against 15 bacteria at a concentration as low as 2.5 mg/mL (Kosani et al. 2016). *Phellinus linteus* extracts showed potent antiinfluenza virus activity. Five phenolic mushroom extracts from *Shiitake*, *Flammulina velutipes*, *A. bisporus*, and Brazilian mushrooms showed varying antibacterial activity (Zhou et al. 2024). Mushroom‐derived phenolic acids, including 2,4‐dihydroxybenzoic, protocatechuic, vanillic, and p‐coumaric acids, demonstrate potent antimicrobial activity against a wide range of bacteria, both Gram‐positive and Gram‐negative, according to recent research. Furthermore, acids like ferulic, caffeic, syringic, ellagic, and chlorogenic also display antimicrobial promise. The study underscores the critical need to elucidate how these compounds work to effectively combat the growing problem of bacteria resistant to multiple antibiotics (Bach et al. 2019).

#### 3.3.2 Anti‐Inflammatory Activity

Anti‐inflammatory substances are essential for preventing excessive inflammation, which is typically regulated by immune cells and can cause damage if there is an overreaction (Jayasuriya et al. 2020). An overactive inflammatory response, triggered by the production of cytokines, chemokines, and prostaglandins, can lead to chronic inflammation, a condition that is essential for healing and combating infections, ultimately contributing to degenerative diseases (Megha et al. 2020).

Polyphenols in mushrooms possess anti‐inflammatory properties by reducing the production of inflammatory mediators (Yu et al. 2025). The ethanol extract of *Morchella esculenta* mycelium inhibits inflammation in rats, while extracts from *G. lucidum* demonstrate anti‐inflammatory effects in carrageenan‐ and formalin‐induced chronic inflammatory models in mice. Additionally, the chloroform extract from *G. lucidum* exhibits significant anti‐inflammatory properties (Anusiya et al. 2021).

A study found that pyrotriol, derived from extracts of *Lactarius deliciosus*, *Cantharellus cibarius*, and *A. bisporus*, demonstrated significant anti‐inflammatory effects (Zhou et al. 2024). Furthermore, phenolic‐rich extracts of *P. eryngii* exhibited both anti‐inflammatory and anticolon cancer properties (Zhou et al. 2024). Research indicates that phenolic extracts from *A. bisporus*, *Boletus impolitus*, *Macrolepiota procera*, and *P. ostreatus* exhibit significant anti‐inflammatory properties by inhibiting the production of nitric oxide. Notably, the extract from *P. ostreatus* demonstrated the most substantial decrease in nitric oxide levels, likely attributed to its cinnamic acid composition (Sharma et al. 2024).

Ando San, an extract derived from Basidiomycetes mushrooms, shows promise in the management of inflammatory bowel diseases like ulcerative colitis and Crohn's disease. Its immunomodulatory and anti‐inflammatory characteristics have been observed to reduce plasma levels of inflammatory markers in patients, indicating its capacity to modulate immune responses and alleviate inflammation‐related symptoms. The study also reported improvements in clinical symptoms and overall quality of life for patients, including decreased abdominal pain and frequency of bowel movements. This suggests that the mushroom extract not only reduces inflammation but also enhances daily functioning and overall well‐being (Therkelsen et al. 2016).

#### 3.3.3 Antioxidant Activity

Mushroom extracts are increasingly utilized as a synthetic source of antioxidants in the pharmaceutical and food industries, contributing biologically active ingredients to various products. The potency of an extract's antioxidant properties is closely linked to the presence of phenolic compounds in mushrooms. Research has identified a strong positive correlation between the antioxidant activity of different mushroom varieties and their phenolic content, likely due to the effective scavenging of hydroxyl radicals by these compounds (Gopal 2021).

Their extracts are increasingly used in food and medicine due to their antioxidant activity, which is influenced by polyphenol levels and can vary with different extraction solvents. *Melanoleuca stridula* and *Melanoleuca cognata* were tested for antioxidant activity using the DPPH assay, revealing the highest activity in water, methanol, and ethyl acetate extracts (Babak et al. 2019; Garrab et al. 2019). A study conducted in Turkey confirmed the relationship between phenol content and antioxidant activity in ethanol extracts of *Polyporus pinicola* and *Polyporus volvatus* mushrooms, revealing high antioxidant activity under similar conditions (Orhan 2022). Additionally, a study of 43 common mushrooms in China discovered that all varieties exhibited antioxidant activity due to the presence of phenolic compounds (Islam et al. 2016). Their phenolic compounds have various antioxidant mechanisms, including electron donors, neutralizing free radicals, maintaining cellular antioxidant effects, chelating metal elements, inhibiting reactive oxygen species, and inhibiting free radical formation through enzymes (Fogarasi et al. 2024).

### 3.4 Antidiabetic Activity

Diabetes mellitus is a chronic metabolic disorder characterized by glucose metabolism dysfunctions, causing continuous blood glucose elevation, leading to long‐term microvascular complications like retinopathy, cardiomyopathy, neuropathy, and nephropathy (Zakir et al. 2023). Diabetes development is influenced by impaired glucose tolerance, gut microbiota disturbance, insulin resistance, and increased fasting blood glucose, and targeted therapies targeting these factors could significantly improve diabetes management (Kelly and Neary 2020). A study found that exopolysaccharides from *Pleurotus geesteranus* have a hypoglycemic effect, repairing pancreatic‐cell damage caused by streptozotocin and acting as an insulin‐like factor to reduce plasma glucose levels (17.1%) (Song et al. 2018). Research into the antidiabetic effects of *Grifola frondosa* polysaccharides showed that high doses caused weight gain in mice, whereas low doses effectively reduced elevated fasting blood glucose levels. Moreover, the low‐dose treatment helped sustain normal blood parameters and significantly lessened inflammatory responses in the liver and kidneys (Chen et al. 2019).

A study on three polysaccharides extracted from the fruiting bodies, caps, and petioles of Suillellus luridus demonstrated promising antidiabetic effects in streptozotocin (STZ)‐induced diabetic mice. These effects included weight loss, increased insulin levels, and enhanced antioxidant enzyme activity (Liu et al. 2022). Studies have shown that *P. eryngii* SI‐04 polysaccharides and *Cordyceps taii* polysaccharides have significant antidiabetic and hypoglycemic properties in mice with STZ‐induced diabetic nephropathy and weight gain, respectively (Liu et al. 2019; Zhang et al. 2018). A study examining six types of mushroom fruiting body polysaccharides found that all types reduced insulin resistance, fat deposition, blood glucose levels, and protected pancreatic islets from high blood glucose damage, using high‐fatty acid and glucose‐induced hepatocytes and a genetically diabetic‐obese (db/db) diabetes mouse model (Liu et al. 2019).

#### 3.4.1 Antitumor Activity

Mushroom extracts are a source of potential antitumor compounds that research indicates may aid in cancer control by hindering cell proliferation and expansion. Studies have identified anticancer phenolic compounds in mushrooms, such as *Albatrellus confluens*, which have shown inhibitory effects on human gastric and ovarian cancer cell lines (Zhou et al. 2024). *Auricularia polytricha* has demonstrated efficacy against colon (COLO‐205), breast (MCF‐7), and kidney (ACHN) cancer cell lines. Furthermore, *Inonotus obliquus*, *G. lucidum* , and *Coprinus atramentarius* have shown anticancer properties, targeting leukemia, lung cancer, and colon cancer cell lines, respectively (Arora et al. 2013).

*Pleurotus ramous* ethyl acetate, methanol, and aqueous extracts showed inhibitory activity on the Dalton's Lymphoma Ascites (DLA) and esophageal adenocarcinoma (EAC) cell lines in rats, with the highest antitumor effect observed in ethyl acetate extracts. A study on rats using *G. lucidum* 's antitumor properties found that methanol and aqueous extracts effectively inhibit tumor growth using an EAC cell line‐induced solid tumor model (Sasikumar et al. 2023). Another study revealed that polysaccharides derived from the mycelium and fruiting bodies of *Pleurotus tuber‐regium* effectively suppressed solid tumor development in rats (Sivanesan et al. 2022).

Flavonoids, a class of phytonutrients present in mushrooms, exhibit notable anticancer effects. They function by inhibiting cell proliferation, inducing apoptosis, and suppressing tumor invasion, while also providing antioxidant benefits. These phytonutrients contribute to a stronger immune response and lower the risk of various cancers, positioning them as valuable components in both cancer prevention and treatment approaches (Camilleri et al. 2024). *Agrocybe aegerita* has been shown to inhibit angiogenesis, a key process in tumor growth, by reducing the production of vascular endothelial growth factor and basic fibroblast growth factor. This suggests it could potentially enhance the therapeutic effects of conventional cancer treatments. Furthermore, the phenolic compounds identified in the water extract of *A. aegerita*, including ferulic, chlorogenic, protocatechuic, gallic, and erucic acids, exhibit significant antitumor activity, indicating potential applications in both tumor prevention and treatment (Abotaleb et al. 2020). Research investigating the antiproliferative effects of *Hypsizygus tessellatus* and *Flammulina velutipes* caps on the MCF‐7 and MDA‐MB‐231 breast cancer cell lines revealed that extracts from both mushroom species exhibited significant antiproliferative activity. The researchers proposed that the hydroxyl groups present in the compounds might interact with mitochondrial cytochrome P450 enzymes (Ukaegbu et al. 2019).

#### 3.4.2 Antiobesity Activity

Obesity is a complex condition marked by excessive fat accumulation, which elevates the long‐term risk of developing diabetes, hypertension, and hyperlipidemia (Chandrasekaran and Weiskirchen 2024). Research indicates that incorporating prebiotics or dietary fiber into the diet may help mitigate the effects of obesity caused by diet. A study investigated the impact of *P. eryngii* polysaccharides (PEP) on body weight and lipid metabolism in mice on a high‐fat diet. The findings showed that PEP supplementation inhibited lipid absorption but also led to increased body weight and mesenteric fat accumulation. However, PEP also enhanced LDL receptor expression, facilitating LDL‐C uptake and reducing serum total cholesterol levels (Nakahara et al. 2019; Zhao et al. 2023).

Research demonstrated that dietary intervention with *Dictyophora indusiata* polysaccharide reversed changes related to high‐fat diet‐induced obesity in obese mice. This resulted in weight loss, reduced lipid accumulation, and a significant decrease in the expression of genes associated with lipid metabolism (Kanwal et al. 2020). Researchers discovered that *Pleurotus geesteranus* exopolysaccharide significantly reduced TC and TG levels in diabetic mice by 18.8% and 12.0%, while also lowering LDL‐C, HDL‐C, and atherogenic index (Duobin et al. 2013). A study demonstrated that *Ganoderma applanatum* polysaccharides can effectively reduce lipid levels in obese rats by reducing food intake and delayed weight gain (Qin et al. 2024). Researchers discovered that *Cordyceps taii* polysaccharides can improve lipid disorders in diabetic mice by affecting lipid metabolism parameters like TC, TG, LDL‐C, and HDL (Liu et al. 2019).

#### 3.4.3 Mushrooms as Functional Foods

Functional foods, such as mushrooms, offer health benefits beyond basic nutrition, protecting against chronic diseases and can be included in various diets due to their medicinal properties and culinary value (Ma et al. 2018). A study found that adding *A. bisporus* extract and dates to bread can improve protein, iron, and nutrient content, reducing anemia and malnutrition. Additionally, adding a water‐alcoholic extract of *A. blazei* mushrooms to milk enhances antioxidant activity and prevents lipid oxidation (Vital et al. 2017).

Mushrooms are increasingly being added to grains to boost their phenolic content, providing antioxidants and antidiabetic benefits. This strategy improves the nutritional value of staple grains and caters to growing consumer demand for functional, health‐promoting foods, thereby promoting health and wellness (Stoffel et al. 2019). A study on puffed snacks found that adding mushrooms like *A. bisporus*, *L. edodes*, and *Boletus edulis* powder significantly improved their antioxidant and antihyperglycemic activity (Na et al. 2023).

Two studies have shown that incorporating mushrooms into everyday food products can improve their nutritional properties and phenolic content, addressing the growing demand for healthier food options. The first study focused on adding specific mushroom varieties to cereal bars, enhancing their antioxidant levels. This has been linked to better cardiovascular health and reduced oxidative stress. The cereal bars provided essential nutrients like proteins and dietary fiber, as well as functional bioactive compounds, making them ideal snacks for health‐conscious individuals (Rosicler et al. 2021; Zhou et al. 2024).

Incorporating mushroom extracts into muffins has been shown to boost both their phenolic content and sensory appeal, according to research. Mushrooms, abundant in bioactive compounds like polysaccharides, vitamins, and antioxidants, enhance the nutritional and functional attributes of baked goods. These enriched muffins offer a well‐rounded nutrient profile, increasing dietary fiber, protein, and antioxidants while maintaining desirable taste, texture, and visual characteristics. The improved sensory qualities make them a convenient option for those seeking nutritious, high‐quality foods without compromising flavor (Singh et al. 2025). Furthermore, the growing trend of including functional ingredients such as mushrooms in everyday foods reflects a move towards enhancing overall dietary quality. With their low‐calorie yet nutrient‐dense composition, mushrooms are a good fit for modern plant‐based diets. Integrating them into common products like muffins and cereal bars enables manufacturers to develop items that supply essential nutrients, offer extra health benefits, and provide convenient, flavorful choices (Kumar et al. 2021).

Mushrooms contain biologically active substances that are widely used in health products, particularly for cognitive support. *H. erinaceus* (lion's mane mushroom) extract is known for its neuroprotective properties, helping prevent neurodegenerative diseases by promoting neuron differentiation and survival through its proteoglycan component. Its bioactive compounds stimulate nerve growth and enhance brain function, making it a key ingredient in cognitive health supplements aimed at improving memory retention and overall neurological well‐being (Szućko‐Kociuba et al. 2023). *G. lucidum* (reishi mushroom) is a powerful health supplement with anti‐inflammatory and antioxidant properties that help prevent alcohol‐induced liver damage, regulate intestinal microbiota, and strengthen immune defense through its polysaccharides. It also enhances immune responses and improves resistance to infections (Ahmad et al. 2023; Guo et al. 2022). *Trametes versicolor* (turkey tail mushroom) supports cancer therapy by boosting immune function with polysaccharide‐K (PSK) and polysaccharide peptide (PSP), complementing chemotherapy (Habtemariam 2020). *Lentinula edodes* (shiitake mushroom) aids cardiovascular health by lowering cholesterol and regulating blood pressure due to eritadenine. Additionally, mushroom‐derived beta‐glucans, prebiotic fibers, and enzymes promote gut health by balancing the microbiome and supporting digestion (Jafari et al. 2023). *Cordyceps sinensis* enhances oxygen uptake and reduces oxidative stress, making it valuable for energy and inflammation control (Sen et al. 2023). In skincare, *Tremella fuciformis* (snow mushroom) hydrates and improves skin elasticity, offering antiaging benefits. With their diverse therapeutic applications, mushrooms serve as essential components in functional foods, nutraceuticals, and pharmaceuticals (Paterska et al. 2024).

### 3.5 Applications of Mushrooms in Industry

#### 3.5.1 Mushrooms as Cosmetics Agents

The cosmetics industry is highly interested in the bioactive compounds found in mushrooms, including phenolic compounds, terpenoids, polysaccharides, and other phytochemicals, due to their potential therapeutic benefits. These substances are utilized in treatments for hyperpigmentation and skin lightening products, primarily through their ability to inhibit tyrosinase. Additionally, they contribute to the firmness and hydration of the skin, creating a more youthful appearance. A wide range of commercially available products, such as antiaging, antifade, lifting, cleansing, hydrating, and moisturizing gels and creams, incorporate mushroom extracts (Rukhsar et al. 2025).

Research indicates that mushroom extracts provide notable cosmetic benefits, addressing skin issues like pigmentation, aging, and wrinkles. *Tremella fuciformis* polysaccharide effectively reduces dark spots and improves skin hydration. *Matsutake* mycelium extract helps minimize elastase activity, elastin breakdown, and wrinkle formation linked to skin aging. Furthermore, three ergosterols from *Grifola frondosa* mycelium inhibit cyclooxygenase 2, a key antiaging enzyme (Paterska et al. 2024). Tyrosinase inhibition is the primary method for inhibiting pigmentation, as it primarily regulates the formation of melanins in organisms. Studies have shown that *Phellinus igniarius* fruiting bodies and *Flammulina velutipes* extract can inhibit tyrosinase activity and melanin synthesis in melanoma cells. A study found that *G. lucidum* , a basidiomycete, has the strongest inhibitory effect on tyrosinase activity, making it widely used in cosmeceutical products (Trepa et al. 2024).

Kitozyme, a Belgian company, has developed patents for purifying glucans from mushrooms, which are used in cosmetics under trade names VITIPURE, ZENVIVO, VELSAN, and Vage Stop. These glucans have potential biological benefits, including antiaging, anti‐microbial, antiirritation, and anti‐oxidant properties (Roszczyk et al. 2022; Vetter 2023). These substances, used in cosmetics like haircare, lipstick, nail makeup, deodorants, and oral care products, have various functions like texturing, wrinkle reduction, skin barrier improvement, elasticity, firmness, and odor control. Alpha Environmental (USA) and MMP (USA) offer moisturizing products like hyaluronic acid and Vegeluron Gel, while Greentech's Biomoduline, a lentinan concentrate from *L. edodes*, has immunostimulant and antiaging properties. Both products are derived from mushrooms (Paterska et al. 2024).

#### 3.5.2 Mushrooms as Food Packaging Agents

Chitosan, a significant component of mushroom cell walls, finds widespread use in food packaging due to its variable purity, molecular weight, salt forms, and viscosity. However, commercial production of chitosan mainly relies on processing shellfish waste (shrimp, crab, and lobster) with strong alkalis at high temperatures (Flórez et al. 2022). Chitin and chitosan production from fungal sources is gaining attention due to their advantages like homogenous polymer length, high deacetylation degree, and solubility (Crognale et al. 2022). Zygomycetous fungi like *Absidia coerulea, Benjaminiella poitrasii, Cunninghamella elegans, Gongrenella butleri, Mucor rouxii, Mucor racemosus*, and *Rhizopus oryzae* have been extensively studied. The isolation of chitosan from a few edible basidiomycetous fungi such as *A. bisporus, L. edodes*, and *Pleurotus sajor‐caju* was also reported (Ghormade et al. 2017). MycoTech industries have explored various organisms like *Aspergillus niger, Penicillium chrysogenum, Saccharomyces cerevisiae*, and other wine yeasts for chitosan production (Ghormade et al. 2017). Fungal chitosan is currently only available from Kitozyme in Belgium. Research suggests using mycelium waste from fungi growing plants or fermentation processes for chitin and chitosan, providing a stable, nonseasonal raw material with more consistent properties than shellfish waste (Pellis et al. 2022).

Ethylene‐coated chitosan films show promise in food packaging due to their antimicrobial properties. DuPont's (USA) ethylene film is effective in preventing chitosan release and demonstrates strong activity against bacteria such as *Escherichia coli* O157:H7 and *Listeria monocytogenes* in beef and chicken (Khubiev et al. 2023). Chitosan was blended with ethylene methylacrylate copolymer ionomer and ethylene vinyl acetate copolymer to develop antimicrobial extruded films with potential applications across multiple industries. Additionally, polylactides and chitosan‐based materials were evaluated for cheese packaging, demonstrating excellent oxygen permeability and antimicrobial properties. These characteristics make them well‐suited for medium‐ and high‐barrier packaging with low water vapor transmission rates (Luzi et al. 2019).

#### 3.5.3 Mushrooms as Pharmaceutical Raw Materials

Medicinal mushrooms have played a vital role in medicine for over 5000 years, highly valued by ancient Chinese and Egyptian cultures. Today, they remain essential in herbal remedies, tonics, and traditional medicine, recognized for their antioxidant, anti‐inflammatory, and immune‐boosting benefits (Nand et al. 2021). Active compounds in specific mushroom species have been shown to reduce the risk of neurodegenerative diseases. A 16‐week Japanese study revealed that consuming a 250 mg tablet of *Hericium erinaceus* extract three times daily led to a significant improvement in cognitive function compared to the control group (Brandalise et al. 2023). Research has shown that bioactive mushrooms hold considerable promise in cancer treatment, making up 16% of all diseases addressed in clinical studies. These findings highlight their potential role in therapeutic applications for cancer care (Panda et al. 2022). A Taiwanese clinical trial revealed that *Antrodia cinnamomea* lowered cancer mortality rates but did not enhance prognosis or affect platelet count, highlighting its potential role in holistic cancer treatments (Zhang et al. 2022).

Clinical trials have validated the immunomodulatory and anticancer effects of mushroom polysaccharides, which have garnered significant interest in the food and pharmaceutical industries. Their ability to trigger apoptosis, suppress tumor cell growth, and enhance immune response underscores their potential in therapeutic applications (Sivanesan et al. 2022). Lentinan, a polysaccharide from *L. edodes*, has been shown to be effective in treating various types of cancer due to its immunostimulatory and cytotoxic properties, activating natural killer cells, boosting macrophage and cytotoxic T cell proliferation, and triggering nonspecific immunological responses (Park 2022).

Ageritin, derived from *Agrocybe aegerita*, has shown cytotoxic properties against certain tumor cell lines (Rotondo et al. 2022), while Eryngitins from *P. eryngii* show no cytotoxicity to human HUVEC cells. Ribotoxin‐like proteins in mushrooms, believed to defend against viruses, molds, and predators, may have potential biomedicine applications as therapeutic agents or biopesticides, but further research is needed (Ragucci et al. 2021).

#### 3.5.4 Mushrooms for Food Product Development

The rich flavor, nutritional benefits, and low allergen content of mushrooms have contributed to their increasing use in meat products, particularly meat analogs, where they present significant commercial potential (Amara and El‐Baky 2023). One study successfully developed a mushroom‐based meat analog by combining edible mushrooms such as *Coprinus comatus*, *L. edodes*, and *P. ostreatus* with soybean protein isolate through a thermos‐extrusion process. The resulting product demonstrated a desirable sensory and textural profile, making it a promising alternative in the food industry (Zhou et al. 2024).

Fresh mushrooms, due to their high moisture content, require immediate consumption or preservation, with drying being a practical and cost‐effective method for long‐term storage. Studies have shown that incorporating powdered mushrooms into bakery products enhances their nutritional value, with optimal sensory properties achieved at an inclusion rate of 4%–10% (Castellanos‐Reyes et al. 2021; Moutia et al. 2024). Additionally, functional breads containing 10% *P. eryngii* exhibited superior sensory characteristics compared to those without mushrooms, despite some alterations in color, odor, and aroma (Zhou et al. 2024). Research also demonstrated that adding *L. edodes* mushroom powder to sweet cereal bars resulted in a final product with excellent textural and sensory qualities (Rosicler et al. 2021). Moreover, a study developed a nutritious snack using *P. eryngii* mushrooms, highlighting their high nutritional and biological value. Sensory evaluations confirmed acceptable product quality, reinforcing the shift toward healthier food choices and emphasizing the need for further research to optimize mushroom dosages (Amerikanou et al. 2023).

#### 3.5.5 Mushrooms as Food Fermenters

Fermentation is a vital process in the food industry that generates biologically active substances from raw materials and fermentation products, using methods like boiling, milling, or sprouting to counteract antinutritional effects (Asensio‐Grau et al. 2020). Solid‐state fermentation, a faster, less expensive, and environmentally friendly method, is more widely used in the food industry than liquid‐state fermentation, which is primarily used by mushrooms for degrading lignocellulosic materials (Bamidele et al. 2025).

Researchers improved the nutritional value and digestibility of lentil flour by fermenting it with *P. ostreatus*, leading to a 6% reduction in carbohydrate content, an 18.5% increase in protein, and a 53% boost in phenolic compounds. These enhancements result from various factors, including carbohydrate bioconversion into protein, nitrogen loss prevention, increased unicellular protein biomass, and the production of extracellular fungal enzymes during growth and bioconversion (Asensio‐Grau et al. 2020). Mushrooms enhance the presence of phenolic compounds in fermented foods through mycelium synthesis, amino acid deamination, and the secretion of phenol oxidases, which break down lignocellulosic materials into nutrient substrates. Research indicates that fermentation enzymes can degrade seeds and starch, releasing phenolic compounds while simultaneously increasing the hydrolyzed protein fraction in fermented flours through mushroom‐induced lytic mechanisms (Kumla et al. 2020).

A study found that fermenting *P. ostreatus* significantly enhances antioxidant activity, total phenolic compounds, protein digestibility, and essential amino acids in kidney bean, black bean, and oat flours—even after simulated digestion. These findings suggest that incorporating *P. ostreatus* in fermentation can improve the nutritional value and health benefits of these flours, emphasizing its potential as a natural and sustainable method (Melanouri et al. 2025). Additionally, *P. ostreatus* plays a crucial role in the synthesis of essential amino acids such as tryptophan and lysine in fermented products, enhancing protein digestibility while reducing antinutrient tannins. Furthermore, a study developed functional cookies using fermented oat, black bean, and kidney bean flours, which exhibited lower sugar levels, higher fiber content, increased bioavailable proteins, stronger antioxidant activity, and superior sensory acceptability compared to commercial cookies (Sá and House 2024).

Fermentation of lentil seeds and quinoa flour increases protein but decreases antioxidant activity and total phenolics, potentially due to microbial or enzymatic differences. *P. ostreatus* fermentation also reduces phytic acid in these ingredients (Sánchez‐García et al. 2023). Furthermore, mushroom mycelia fermentation of tofu, particularly with *P. ostreatus*, significantly boosts isoflavone aglycone content, leading to beneficial physiological effects like ACE and α‐glucosidase inhibition, as well as anticancer and anti‐inflammatory properties. *P. ostreatus*‐fermented tofu showed a higher aglycone conversion rate than *L. edodes*‐fermented tofu (89.1% vs. 51.3%). Unlike the more easily absorbed isoflavone glycosides, aglycones' smaller size and lower hydrophilicity may influence their absorption and potential role in cancer prevention (Sawada et al. 2023).

### 3.6 Application of Mushrooms on Environmental Bioremediation

Industrialization, agrochemical use, and urbanization have contributed to soil and water pollution, introducing hazardous waste, heavy metals, and organic contaminants that disrupt ecosystems (Ayilara and Babalola 2023). Traditional metal removal methods are often inefficient or costly, making biotechnology a promising alternative. Bioremediation, an environmentally friendly and cost‐effective approach, utilizes microorganisms to degrade pollutants, particularly heavy metals. While bioremediation is effective for low metal concentrations, it does generate toxic sludge. Despite the significant financial investment required, biotechnology provides a clean and nondisruptive solution (Kuppan et al. 2024).

Mushrooms are effective in bioremediation, reducing soil and water pollution by removing or neutralizing pollutants. They degrade organic and inorganic wastes, transforming them into high‐quality, flavorful, and affordable foods, particularly beneficial for restoring damaged environments (Jasinska 2023; Okuda 2022). Mushrooms, a type of saprophytic fungi, have shown potential for environmental bioremediation. Their myco‐restoration through myco‐filtration, myco‐forestry, mycoremediation, and myco‐pesticides can effectively reclaim defective environments and remove harmful substances from soil, according to recent research (Akpasi et al. 2023). Mushroom‐forming fungi are potent decomposers, secreting strong enzymes due to their aggressive growth and biomass production. They can degrade lignocellulosic materials into human food and act as bio‐sorbents of cyanogenic metals. Alternative fungi and cultivated mushrooms have protein appliances for deteriorating environmental contaminants (Kumla et al. 2020).

Mushrooms are vital in the soil food network, reducing contaminants and providing nutrition to alternative biotas. They degrade and recycle waste, uptake heavy metals through biosorption, and act as biological filters and sorbents. Their abundant hyphae aid in nutrient and heavy metal biosorption, while their rough texture attracts contaminants. Wild mushrooms contain high concentrations of Fe, Hg, As, Cd, Zn, and Pb in their sporocarps (Akpasi et al. 2023). Research highlights mushroom remediation as an effective and sustainable approach for biodegrading persistent pollutants in soil and wastewater through enzyme production. Agricultural pesticide misuse leads to residue accumulation in soil, posing environmental risks. *P. ostreatus* has demonstrated the ability to mineralize insecticides, degrade DDT and malathion, and remove pentachlorophenol, showcasing its potential in environmental cleanup efforts (Ghose and Mitra 2022; Llanaj et al. 2023).

*Pleurotus* species, found worldwide, serve as primary decomposers of hardwood trees and play a vital role in the in situ degradation of xenobiotics, phenolics, and coloring pigments in effluents (Sekan et al. 2019; Torres‐Farradá et al. 2024). Agricultural waste generation—including harvesting, processing, and consumption—frequently leads to disposal challenges. Agro‐industrial units produce substantial amounts of waste, such as rice straw, which is low in protein and digestibility. *Pleurotus* species can enhance cattle feed by colonizing waste and improving digestibility. Laccase, an extracellular phenol oxidase present in various mushrooms, is essential in lignin biodegradation (González‐González et al. 2023). Additionally, the non‐specific oxygenases of *Pleurotus* spp. facilitate the bioconversion of agro‐wastes, including the degradation of chlorinated mono‐aromatics and other xenobiotic compounds. These capabilities are being explored for bioremediation efforts, such as the biodegradation of industrial effluents like dyes (Torres‐Farradá et al. 2024).

Food containing dyes poses environmental risks because factory residues are discharged into rivers and streams. However, *Pleurotus florida* and *Trametes versicolor* have demonstrated effectiveness in dye degradation; *P. florida* produces three lignolytic enzymes that break down lignin, polycyclic aromatic hydrocarbons, polychlorinated biphenyl mixtures, and synthetic dyes (Upadhyay et al. 2024). Furthermore, mushroom hyphae and mycelia can degrade crude oil and heavy metals in polluted soil, converting pollutants into carbon dioxide, water, and potentially biomass. The anthracene degradation rate of *P. ostreatus* depends on factors such as time, contamination level, and fungal treatment. Additionally, *Pleurotus* species can produce industrial enzymes using agro‐waste, and *P. tuberregium* can improve crude oil‐polluted soil, leading to significant environmental benefits (Vaksmaa et al. 2023).

### 3.7 Application of Mushrooms on Bioenergy Production

Mushroom cultivation is a crucial biotechnology that offers nutritious food and functional bioactivities, driving global market growth. Its health‐promoting properties and essential nutrients make it a vital part of the functional food industry. Mushroom cultivation also recycles agricultural and organic waste materials into valuable biomass, converting organic materials like straw and sawdust into nutritious food. This supports circular economy principles by minimizing waste and adding value to discarded resources (Llanaj et al. 2023).

Waste‐to‐fuel concepts are innovative and sustainable methods for repurposing waste materials, such as spent mushroom substrate (SMS), as valuable feedstock for biofuel production. These processes not only manage waste but also contribute to the generation of renewable energy sources, reducing reliance on fossil fuels and lowering greenhouse gas emissions. Key biofuels produced through waste‐to‐fuel technologies include bioethanol, biogas, bio‐hydrogen (bio‐H_2_), bio‐oil, and solid biofuels (Łysakowska and Sobota 2023).

Bioethanol is a renewable liquid fuel produced from the fermentation of sugars or cellulose derived from organic waste, including SMS. This process involves microorganisms breaking down carbohydrates into ethanol, which can be used as an alternative to gasoline in transportation. Biogas, another important biofuel, can be produced from SMS through anaerobic digestion, generating a mixture of methane (CH_4_) and carbon dioxide (CO_2_), which can be used as a renewable energy source for heating, electricity generation, and even as a vehicle fuel. The byproduct of anaerobic digestion, known as digestate, can be used as a nutrient‐rich organic fertilizer, further enhancing the sustainability of the process (Bušić et al. 2018; Mukherjee et al. 2023).

Bio‐hydrogen (Bio‐H_2_) is a clean fuel produced through biological processes that convert organic waste materials into hydrogen gas. It is a sustainable and renewable energy source that addresses the growing demand for cleaner fuels while reducing environmental impact (Kazmi et al. 2024). Spent mushroom substrate (SMS) is a promising feedstock for bio‐hydrogen production, providing carbon and nutrients for microorganisms to generate hydrogen gas under anaerobic conditions. Dark fermentation and photo‐fermentation are the primary biological methods for hydrogen production, with dark fermentation breaking down carbohydrates and photo‐fermentation using light to drive hydrogen production by photosynthetic bacteria. Combining these processes can significantly increase hydrogen yields from organic waste materials (Qu et al. 2022).

Bio‐hydrogen (Bio‐H2) is a clean fuel produced through biological processes, converting organic waste materials into hydrogen gas. It is a sustainable and renewable energy source, meeting the growing demand for cleaner fuels while reducing environmental impact. Spent mushroom substrate (SMS) is an excellent feedstock for bio‐hydrogen production (Mustapha et al. 2025). Two primary biological methods for hydrogen production are dark fermentation and photo‐fermentation. Dark fermentation involves microorganisms breaking down carbohydrates and other organic compounds in SMS, while photo‐fermentation uses light to drive hydrogen production by photosynthetic bacteria. Combining dark fermentation and photo‐fermentation can significantly increase hydrogen yield from organic waste materials, making it a promising approach for converting SMS and other agricultural wastes into bio‐hydrogen sources (Aboelela et al. 2023).

The integration of mushroom cultivation for food and biofuel production can meet global energy and food demands, as it efficiently promotes biofuel yield, improves the economy, and supports the biorefinery approach (Martín et al. 2023). The mushroom industry is addressing the issue of SMS waste accumulation, which could harm the environment, economy, and human health if not properly managed. Innovative technologies are being developed to convert SMS waste into valuable products such as biofertilizers and animal feed. Strict regulations and guidelines are being implemented to minimize its environmental impact. Stakeholders must collaborate to find sustainable solutions, invest in research and development, and take proactive steps towards sustainable SMS waste management to reduce the environmental footprint while improving efficiency and profitability (Llanaj et al. 2023).

## 4 Future Prospects

Mushroom research is poised to revolutionize sectors such as healthcare, pharmaceuticals, and environmental sustainability. The discovery of novel bioactive compounds in mushrooms opens up possibilities for developing new therapies targeting neurodegenerative and cardiovascular diseases, as well as cancer. These compounds could lead to groundbreaking medical treatments that harness mushrooms' therapeutic properties. In the food and nutraceutical industries, mushrooms are emerging as functional foods and sustainable meat substitutes due to their rich nutrient profiles and bioactive compounds. Their ability to manage chronic conditions like diabetes, hypertension, and high cholesterol positions them as vital components in personalized nutrition and health solutions. Technological advances in extraction and cultivation techniques will further enhance the cosmetic and pharmaceutical applications of mushrooms. Mushrooms' potential in bioremediation for cleaning up heavy metals and pollutants is particularly promising. Their capacity to convert waste into biofuels and other valuable byproducts could transform waste management and contribute to a more sustainable future.

## Author Contributions

**Gebreselema Gebreyohannes:** conceptualization (equal), data curation (equal), formal analysis (equal), project administration (equal), supervision (equal), validation (equal), writing – original draft (equal), writing – review and editing (equal). **Solomon Fitsum:** conceptualization (equal), data curation (equal), formal analysis (equal), writing – original draft (equal). **Desta Berhe Sbhatu:** conceptualization (equal), supervision (equal), writing – review and editing (equal).

## Conflicts of Interest

The authors declare no conflicts of interest.

## Data Availability

The data that support the findings of this study are available from the corresponding author upon reasonable request.
